# Efficacy of continuous versus intermittent subglottic secretion drainage in preventing ventilator-associated pneumonia in patients requiring mechanical ventilation: A single-center randomized controlled trial

**DOI:** 10.18632/oncotarget.24630

**Published:** 2018-03-23

**Authors:** Hiroko Fujimoto, Osamu Yamaguchi, Hajime Hayami, Mika Shimosaka, Sayaka Tsuboi, Mitsunori Sato, Shigeo Takebayashi, Satoshi Morita, Mari Saito, Takahisa Goto, Kiyoyasu Kurahashi

**Affiliations:** ^1^ Division of Critical Care, Yokohama City University Medical Center, Minami-ku, Yokohama, Kanagawa 232-0024, Japan; ^2^ Department of Anesthesiology and Critical Care Medicine, Yokohama City University Graduate School of Medicine, Kanazawa-ku, Yokohama, Kanagawa 236-0004, Japan; ^3^ Radiation Department, Yokohama City University Medical Center, Minami-ku, Yokohama, Kanagawa 232-0024, Japan; ^4^ Department of Biostatistics and Epidemiology, Yokohama City University Graduate School of Medicine, Kanazawa-ku, Yokohama, Kanagawa 236-0004, Japan; ^5^ Department of Anesthesiology and Intensive Care Medicine, International University of Health and Welfare, School of Medicine, Narita, Chiba 286-8686, Japan

**Keywords:** ventilator-associated pneumonia, subglottic secretion drainage, length of mechanical ventilation

## Abstract

**Objective:**

Aspiration of subglottic secretion is a widely used intervention to prevent ventilator-associated pneumonia (VAP). This study aimed to compare the efficacy of continuous and intermittent subglottic secretion drainage (SSD) in preventing VAP.

**Methods:**

A single-center randomized controlled trial was conducted on adult postoperative patients who were expected to undergo mechanical ventilation for more than 48 hours. Primary outcome measure was incidence of VAP and secondary outcome measures were length of mechanical ventilation and intensive-care unit (ICU) stay.

**Results:**

Fifty-nine patients received continuous SSD, while 60 patients received intermittent SSD. Of these 119 patients, 88 (74%) were excluded and 15 and 16 patients were allocated to receive continuous and intermittent SSD, respectively. VAP was detected in 4 (26.7%) and 7 (43.8%) patients in the continuous and intermittent groups, respectively, (p=0.320). The length of mechanical ventilation was significantly shorter (p=0.034) in the continuous group (99.5±47.1 h) than in the intermittent group (159.9±94.5 h). The length of ICU stay was also shorter (p=0.0097) in the continuous group (6.3±2.1 days) than the intermittent group (9.8±4.8 days).

**Conclusions:**

Although continuous SSD did not reduce the incidence of VAP, it reduced the length of mechanical ventilation and ICU stay when compared to intermittent SSD.

## INTRODUCTION

Ventilator-associated pneumonia (VAP) is a common and serious complication of mechanical ventilation. Despite the use of a wide range of preventive measures, the incidence of VAP in the intensive care units (ICU) ranges from 9% to 27% [1], and the crude mortality rate ranges from 25% to 50% [2–4]. Safdar et al. showed that VAP was associated with a mean ICU length of stay of 6.10 additional days (95 % CI, 5.32–6.87) [5]. The study found that VAP also increased healthcare costs [5, 6]. Muscedere et al. reported an amount of 11,450 Canadian dollars as the additional cost of VAP per case [6].

Many studies have shown that the accumulation of subglottic secretions above the endotracheal cuff plays an important role in the pathogenesis of VAP [1, 2, 7]. Therefore, drainage of the subglottic secretions to prevent VAP was attempted using a separate dorsal lumen that opens above the endotracheal tube cuff [8–16]. Subglottic secretion drainage (SSD) has been shown to be associated with a lower incidence of VAP in previous meta-analyses [17–19]. In a study by Wang et al. [17], both continuous and intermittent SSD reduced the incidence of VAP. However, previous studies have not directly compared the efficacy of intermittent SSD to that of continuous SSD. Continuous SSD is considered to be superior to intermittent SSD in terms of reducing the incidence of influx of subglottic secretion into the trachea. However, continuous SSD has a risk to cause a herniation of tracheal mucosa into the subglottic suction port, and may lead to either mucosal injury [20] or aspiration insufficiency [21].

The aim of this study was to compare the efficacy of continuous and intermittent SSD in the prevention of VAP.

## RESULTS

**Figure 1:**
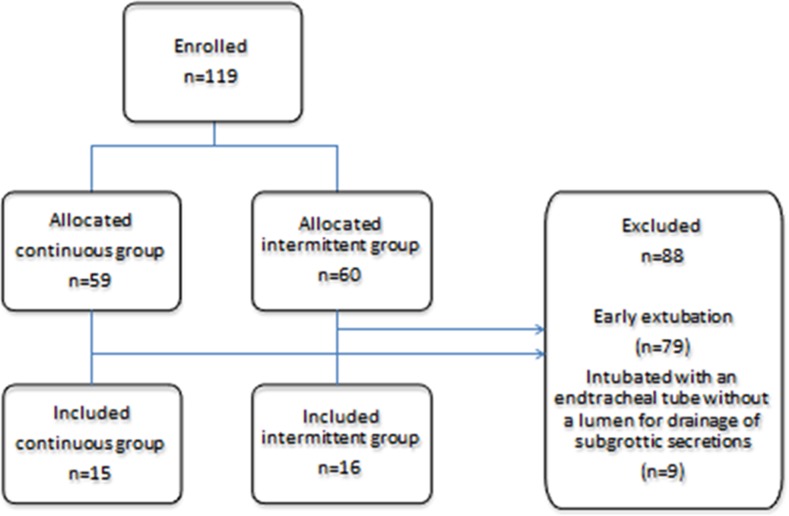
Consort flow diagram for the present study

**Figure 2:**
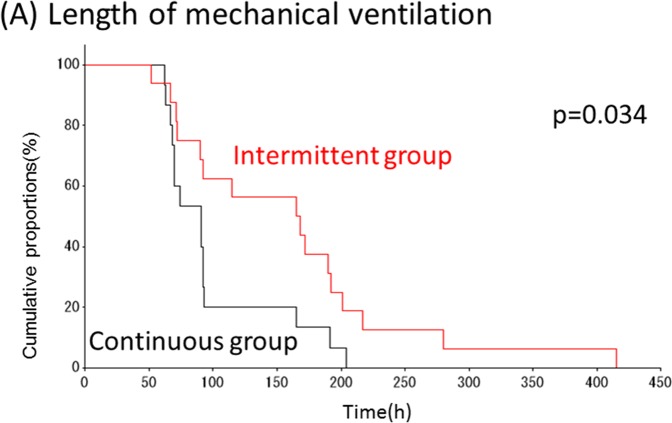

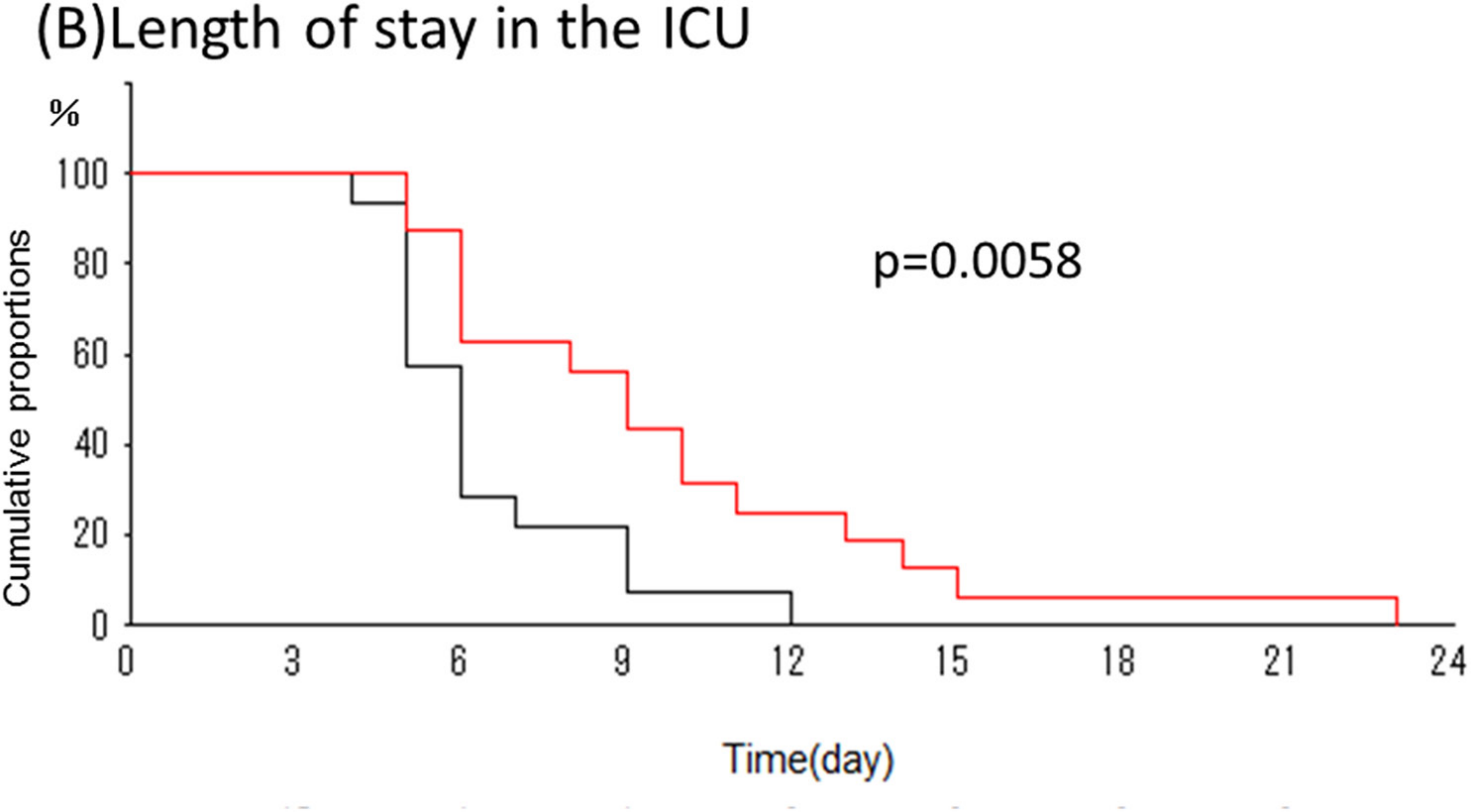
Cumulative probability of outcomes in two groups Cumulative probability values for mechanical ventilation **(A)** and ICU stay **(B)** were both significantly shorter in the continuous group compared with the intermittent group.

During the study period, 59 patients were randomized to the continuous group, and 60 to the intermittent group. Of these 119 patients, 88 (74 %) were excluded from the study for the following reasons: early extubation (79 patients) or intubation with an endotracheal tube without a lumen for drainage of subglottic secretions (9 patients). Of the remaining 31 patients in the study, 15 were in the continuous SSD group and 16 in the intermittent SSD group (Figure 1). Patients in both groups were similar in regard to their demographic characteristics, surgical procedures, and the severity of illness (Table 1). In this study, VAP, including ventilator-associated tracheobronchitis (VAT), was detected in 11 of the 31 patients (Table 2); 4 (26.7%) were in the continuous group and 7 (43.8%) in the intermittent group. No statistically significant differences were observed in the incidence of VAP/VAT between the two groups (Table 3). The length of mechanical ventilation was shorter in the continuous group (99.5±47.1 h) than in the intermittent group (159.9±94.5 h) (p=0.034; Figure 2A). The length of ICU stay was also shorter in the continuous group (6.3±2.1 days) than in the intermittent group (9.8±4.8 days) (p=0.0097; Figure 2B). No significant differences were observed in hospital mortality rates or the length of hospital stay, and no complications related to SSD, such as mucosal injury, were noted.

**Table 1:** Patient characteristics

Characteristics	Continuous group (n=15)	Intermittent group (n=16)	P value
Age	68.1±9.4	70.9±8.9	0.313
Sex			0.809
Male	10 (66.7 %)	10 (62.5 %)	
Female	5 (33.3 %)	6 (37.5 %)	
Smoker (n)	10(66.7%)	11(68.8%)	0.901
Preoperative total protein (g/dL)	7.0±0.8	7.0±0.6	0.944
Preoperative total lymphocyte count less than 1200 /uL (n)	6	6	1.000
Type of surgery (n)			0.474
Esophageal surgery	3(20.0 %)	5(31.2 %)	
Cardiovascular surgery	12(80.0%)	11(68.8%)	
Operation time (min)	496±165	479±119	0.921
Intraoperative fluid balance (ml)	7476±5186	7278±3148	0.649
APACHE II score	14.6±5.7	14.8±3.8	0.874
Average SOFA score	6.0±2.1	5.3±3.0	0.373

APACHE = Acute Physiology and Chronic Health Evaluation, SOFA = Sequential Organ Failure Assessment.

**Table 2:** Details of patients who developed VAP/VAT

Age, sex	Group	Type of surgery	VAP or VAT	PaO_2_/FiO_2_ ratio	Time to VAP/ VAT(day)	Pathogenesis	length of ICU stay(day)	length of mechanical ventilation(h)
68/F	Continuous	Esophageal surgery	VAT	265.5	7	Streptococcus pneumoniae	9	165
83/M	Intermittent	Vascular surgery	VAT	211.3	2	Pseudomonas aeruginosa	23	415
76/M	Intermittent	Esophageal surgery	VAT	257.2	7	Haemophilus influenza	9	168
70/F	Continuous	Cardiac surgery	VAP	180.8	4	Pseudomonas aeruginosa	4	70
67/M	Continuous	Esophageal surgery	VAP	262.6	7	Haemophilus influenza	9	191
74/M	Intermittent	Vascular surgery	VAP	229	5	Ppseudomonas aeruginosa	13	192
75/M	Continuous	Vascular surgery	VAT	214.5	3	Pseudomonas aeruginosa	12	204
70/F	Intermittent	Cardiac surgery	VAT	211.3	6	Enterobactor cloacae	15	280
74/F	Intermittent	Vascular surgery	VAP	168.1	4	Serratia marcessece	8	115
79/F	Intermittent	Cardiac surgery	VAP	255	5	Enterobactor cloacae	14	201
64/M	Intermittent	Esophageal surgery	VAT	205.6	4	MRSA	10	172

^*^Median (IQR).

VAP = ventilator-associated pneumonia, VAT = ventilator-associated tracheobronchitis, MRSA = Methicillin-resistant *Staphylococcus aureus*.

**Table 3:** Clinical outcomes of the patients receiving mechanical ventilation for >48 h

	Continuous group (n=15)	Intermittent group (n=16)	P value
VAP Incidence rate (%)	4 (26.7%)	7 (43.8%)	0.320
Length of mechanical ventilation (h)^*^	91.0 (68.0–93.0)	166.5 (81.0–196.5)	0.034
Length of ICU stay (days)^*^	6.0 (5.0–7.0)	9.0 (6.0–12.0)	0.0058
Length of hospital stay (days)^*^	30.0 (19.0–50.0)	38.0 (29.0–74.0)	0.0818
Time to VAP (days)^*^	7.0 (7.0- )	7.0 (5.0- )	0.777
Mortality	2	2	1.00

^*^Median (IQR).

VAP = ventilator-associated pneumonia.

## DISCUSSION

Previous meta-analyses [17–19] have shown that SSD was associated with a lower incidence of VAP; however, these meta-analyses included patients treated with both continuous and intermittent SSD. The present study was the first study that compared the effects of continuous SSD with that of intermittent SSD on the incidence of VAP. We found a trend toward decreased incidence of VAP in the continuous SSD group when compared with that of the intermittent SSD (26.7% vs. 43.8%); however, this difference did not reach statistical significance. We postulated that this was caused by a lack of power because a considerable proportion of patients were excluded from the study mainly due to early extubation. Further study with a better protocol such as excluding fewer patients is warranted. Nevertheless, demographic and preoperative conditions including age, sex, percentage of smokers, or nutrition impairment assessed by total protein and lymphocyte count were not different between the groups. In addition, factors during the surgery including type of surgery, intraoperative fluid balances, or operation time were not different and the severity of the patients’ condition at the time admitted to ICU (as determined by the Acute Physiology and Chronic Health Evaluation (APACHE) II score) or during the stay in ICU (average SOFA score) were not different between the groups.

The incidence of VAP or VAT was higher than that in the previous reports [1, 21]. There were no standard diagnostic criteria for VAP/VAT when the present study was conducted in 2010 and we defined microbiological criteria for VAP/VAT as polymorphonuclear leukocytes stained by gram staining with or without bacteria in the endotracheal aspirate. Our diagnostic criteria might have been broad resulting in a relatively high incidence of VAP/VAT in either group.

Secondary outcomes including length of mechanical ventilation and length of ICU stay demonstrated significantly better outcomes for each variable in the continuous SSD group when compared to the intermittent group. This was a striking result of the present study despite a considerable number of patients being excluded from the study. These facts also encourage us to further plan another study with a larger number of patients fulfilling the study criteria.

Dragoumanis et al. reported that continuous SSD failed to aspirate subglottic secretions in about 40% of the patients when herniation of the tracheal mucosa into the subglottic suction port occurred [22]. Another study reported that tracheal injury still occurred following the use of continuous SSD despite efforts being implemented to avoid herniation by using a wall suction unit with a very low negative pressure (maximum 20 cm H_2_O) [20]. In contrast to previous studies that used wall suction units for continuous SSD [9, 14, 15], we used the HAMA^®^ for the continuous group in this study. We observed the tracheal mucosa of a patient who used HAMA^®^ as long as 9 days using a bronchoscope and found no complications such as mucosal injury. For other patients, however, we evaluated the occurrence of complications based on medical records and did not actively evaluate them. Although we did not have any patients with tracheal mucosal injury, we may have missed the occurrence of complications with continuous SSD. Further study is warranted before we conclude the safety of the continuous SSD.

To avoid mucosal injury, we set the suction pressure of HAMA^®^ to −30 cm H_2_O which was lower than that for the intermittent group. Lower negative pressure may be inadequate to aspirate subglottic secretion; however, the incidence of VAP/VAT did not increase with continuous SSD.

The present study has several limitations. First, because many patients were initially excluded from our study, we were able to collect data from only a small cohort of patients. In addition, we found significant differences in the secondary outcomes; however, the number of patients was calculated based on the primary outcome estimates. Further studies utilizing larger patient cohorts are needed. Second, we performed continuous SSD for a maximum of 9 days in the present study. Further studies are therefore required to ensure the safety of continuous SSD over longer periods. Third, the physicians who assessed the results were not blinded to the assignment of the procedure, which potentially introduced bias.

In conclusion, our results showed that continuous SSD reduced the length of mechanical ventilation and length of ICU stay when compared to intermittent SSD. Further studies that enroll large enough cohort of patients are needed to clarify whether continuous SSD reduces the incidence of VAP/VAT when compared with intermittent SSD.

## MATERIALS AND METHODS

This study was approved by the Ethics Committee of Yokohama City University, and written informed consent was obtained from each patient. The study was registered at the UMIN-CTR (University Hospital Medical Information Network-Clinical Trials Registry, ID: UMIN000003553, registered 01 May 2010, URL: https://upload.umin.ac.jp/cgi-open-bin/ctr/ctr.cgi?function=brows&action=brows&recptno=R000004290&type=summary&language=E).

This single-center randomized controlled trial was conducted from May 2010 to March 2011. We studied adult (≥18 years of age) postoperative patients who were expected to undergo mechanical ventilation for >48 h. Patients were excluded if they had developed pneumonia at admission or had been intubated anywhere other than in an ICU or operating room. Patients were randomly assigned to receive either continuous aspiration (continuous group) or intermittent manual aspiration (intermittent group). Stratified block randomization was performed by the staff of the Department of Biostatistics and Epidemiology using computer software. We regarded esophagectomy as a stratified factor because most of the patients who underwent surgery in our hospital for esophageal cancer had lost their cough reflex, and thus, required prolonged mechanical ventilation. In the continuous group, we used HAMA^®^ SERVO-DRAIN (type SD-2002, INNOMEDICS, Tokyo, Japan), a low-pressure drainage system. We connected the HAMA^®^ to the subglottic suction port and operated it intermittently at a pressure of −30 cm H_2_O for 20 seconds with an interval of 20 seconds. We chose this setting to prevent mucosal injury. In the intermittent group, subglottic suctioning was delivered manually using a standard wall suction unit at a negative pressure of 100–150 mmHg by nurses every 2 h.

In both groups, standard care to prevent VAP was followed, as stated below:

(1) The patient was maintained in a semirecumbent position (45°) unless contraindicated, and was alternately placed in the right or left semirecumbent position every 2 h. (2) Readiness of enteral nutrition was frequently assessed and was initiated as soon as it became possible. (3) Oral cleaning with 2% povidone-iodine was provided once every 8 h. (4) The intracuff pressure of the tracheal tube was verified every 2 h and maintained at 20 cmH_2_O.

Both groups also received the standard of care which included insulin therapy to maintain their serum glucose level under 180 mg/dL, daily chest radiographic examinations, and bacteriological examinations of airway secretion sampled through the endotracheal tube twice a week. In addition, we performed daily extubation tests including spontaneous breathing test on each patient and each patient was evaluated for ICU indication every day, which standardized the patient outcomes, including length of mechanical ventilation and the length of ICU stay. The follow-up period of the patients was until discharge from the hospital.

The primary outcome was the incidence of VAP including ventilator-associated tracheobronchitis (VAT). Secondary outcome measures included the length of mechanical ventilation and the length of ICU stay.

Diagnostic criteria for VAP/VAT [21] included the following:

One or more findings of elevated body temperature (>38 °C), leukocytosis (>12,000/mL), or leukopenia (<4,000/mL), in addition to purulent endotracheal secretions or an increase in sputum production.

VAP: associated with new and progressive or persistent infiltrates, diagnosed by a radiologist who was blinded to the study group

VAT: associated with no new infiltrates

Gram staining of endotracheal aspirates with polymorphonuclear leukocytes with/without bacteria

### Statistical analysis

Based on a previous study [17], estimated efficacy rates for intermittent and continuous SSD were assumed to be 20% and 55%, respectively. With an alpha error of 0.10 and a beta error of 0.2, total sample size to detect a statistically significant difference between the groups was calculated as 46. The differences in the patients’ characteristics and incidence of VAP between the two groups were compared using Student’s t-test, chi-squared test, or Wilcoxon test. The length of mechanical ventilation and the length of ICU stay were summarized using the Kaplan–Meier method and compared by the log-rank test. We performed a chi-squared test for incidence of VAP and Fisher’s exact test for mortality. Statistical significance was defined as P < 0.05. Statistical analyses were performed using SPSS Ver19 (IBM Corp, Armonk, NY) and SAS 9.3 (SAS institute, Cary, NC).

## Abbreviations

ICU: intensive care unit
SSD: subglottic secretion drainage
VAP: ventilator-associated pneumonia
VAT: ventilator-associated tracheobronchitis.
